# Factor H Autoantibodies and Complement-Mediated Diseases

**DOI:** 10.3389/fimmu.2020.607211

**Published:** 2020-12-15

**Authors:** Yuzhou Zhang, Nicolo Ghiringhelli Borsa, Dingwu Shao, Arthur Dopler, Michael B. Jones, Nicole C. Meyer, Gabriella R. Pitcher, Amanda O. Taylor, Carla M. Nester, Christoph Q. Schmidt, Richard J. H. Smith

**Affiliations:** ^1^ Molecular Otolaryngology and Renal Research Laboratories, University of Iowa, Iowa City, IA, United States; ^2^ Institute of Pharmacology of Natural Products & Clinical Pharmacology, Ulm University, Ulm, Germany

**Keywords:** factor H, autoantibodies, complement, C3 glomerulopathy, atypical hemolytic uremic syndrome, monoclonal gammopathy of renal significance

## Abstract

Factor H (FH), a member of the regulators-of-complement-activation (RCA) family of proteins, circulates in human plasma at concentrations of 180–420 mg/L where it controls the alternative pathway (AP) of complement in the fluid phase and on cell surfaces. When the regulatory function of FH is impaired, complement-mediated tissue injury and inflammation occur, leading to diseases such as atypical hemolytic uremic syndrome (a thrombotic microangiopathy or TMA), C3 glomerulopathy (C3G) and monoclonal gammopathy of renal significance (MGRS). A pathophysiological cause of compromised FH function is the development of autoantibodies to various domains of the FH protein. FH autoantibodies (FHAAs) are identified in 10.9% of patients with aHUS, 3.2% of patients with C3G, and rarely in patients with MGRS. The phenotypic variability of FHAA-mediated disease reflects both the complexity of FH and the epitope specificity of FHAA for select regions of the native protein. In this paper, we have characterized FHAA epitopes in a large cohort of patients diagnosed with TMA, C3G or MGRS. We explore the epitopes recognized by FHAAs in these diseases and the association of FHAAs with the genetic deletion of both copies of the *CFHR1* gene to show how these disease phenotypes are associated with this diverse spectrum of autoantibodies.

## Introduction

Complement factor H (FH), a 155 KDa glycoprotein comprised of 20 short consensus repeat (SCR) domains, circulates in the blood at concentrations of 180–420 mg/L. It functions as the major regulator of the alternative pathway (AP) of complement in the fluid phase and on cell surfaces. Fluid-phase complement regulation is mediated by the four N-terminal SCRs of FH through two different mechanisms—decay accelerating activity (DAA) and co-factor activity (CA). DAA refers to the ability of FH to promote displacement of the Bb fragment of factor B (FB) off C3 convertase through SCR1-2, thereby accelerating the irreversible decay of C3bBb to C3b and Bb. CA refers to the role of FH as a facilitator of factor I (FI)-mediated proteolytic cleavage of C3b to an inactivated form of C3b called iC3b (1). In both scenarios, FH SCR1-4 interacts with the MG ring, CUB domain and TED domain on the C3b molecule (2). On cell surfaces, FH protects host surfaces from complement-mediated damage primarily through the two C-terminal SCRs, which recognize and bind to sialic acids, glycosaminoglycans (GAG), heparins, and a site on the C3b-cleavage fragment C3d (3, 4). These binding events ensure that the DAA function of FH is targeted to host cell surfaces, thereby protecting these cells from indiscriminate complement amplification that might otherwise be associated with surface deposition of C3b (5).

Genetic variation and/or acquired autoantibodies are the two major factors that impair FH function, and therefore are the primary drivers of two complement-mediated renal diseases, atypical hemolytic uremic syndrome (aHUS) and C3 glomerulopathy (C3G). The former, a type of thrombotic microangiopathy (TMA), is characterized by hemolytic anemia, thrombocytopenia and acute renal injury (6). TMA itself is an overarching term used to describe any condition characterized by thrombocytopenia and microangiopathic hemolytic anemia (MAHA) with varying degrees of organ damage in the setting of normal clotting parameters. Although tissue diagnosis, most commonly in the form of a kidney biopsy showing abnormalities in arterioles and capillaries with microvascular thrombosis, is required, TMA is often inferred from the observation of thrombocytopenia and MAHA in the appropriate clinical setting. Complement-mediated aHUS occurs primarily on host cell surfaces and leads to acute vascular endothelial injury and thrombosis. When left untreated, the likelihood of renal failure and mortality are high (7). The second disease associated with impaired FH function, C3G, is a chronic glomerulopathy characterized by predominant C3 deposition in renal glomeruli. Classic findings of glomerulonephritis (hematuria, proteinuria and variable degrees of renal impairment) result from fluid-phase complement dysregulation (8). Renal survival is about 10 years in up to 50% of affected individuals and following transplantation, approximately 50% of patients experience disease recurrence with allograft loss.

FHAAs have been identified as drivers of complement dysregulation in both aHUS and C3G (9). They are more common in aHUS, being detected in ∼10% of patients in European cohorts and up to 50% in an Indian aHUS cohort (10–12). In C3G cohorts, FHAAs are present in ~3% of patients (13, 14).

Interestingly, in aHUS, the presence of FHAAs is often associated with a common genetic variation known as a copy number variation (CNV) in the *CFH-CFHR* genomic region. The *CFH*-*CFHR* gene family includes, in addition to *CFH*, five complement factor H-related (*CFHR*) genes located directly 3’ of the *CFH* gene in the order of *CFHR3, CFHR1, CFHR4, CFHR2*, and *CFHR5*. The *CFHR* genes arose as a result of genomic duplication and because of the high sequence homology, the region is prone to non-allelic homologous recombination, a process that can result in gene deletion, duplication and rearrangement.

Non-allelic homologous recombination gives rise to CNVs. Absence of both copies of *CFHR1* due to homozygous deletion of *CFHR3-CFHR1* (del*CFHR3-1*) or less commonly compound heterozygous deletion of *CFHR3-CFHR1* and *CFHR1-CFHR4* or homozygous deletion of *CFHR1-CFHR4* is associated with an increased relative risk for aHUS as a consequence of the development of FHAAs, referred to as DEAP-HUS, *DE*ficiency of CFHR1 plasma proteins and *A*utoantibody *P*ositive *H*emolytic *U*remic *S*yndrome (15). It is important to note, however, that the increase in relative risk is small as homozygous del*CFHR3-1* is common. About 3% of European-Americans do not have any copies of *CFHR3-CFHR1*, a percentage that varies significantly by ethnic group (16, 17). The mechanism underlying the development of FHAAs in association with FHR1 deficiency is not well understood but may reflect structural differences between FHR1 and the carboxy terminus of FH (18). To date, there has been no such kind of association observed in C3G. In fact, FHAAs identified in C3G patients are frequently associated with the presence of C3 nephritic factors in children and with monoclonal gammopathy of renal significance (MGRS) in adults (13, 14, 19, 20).

Herein, we report the prevalence and immunological features of FHAAs in cohorts of aHUS and C3G patients from the USA.

## Methods

### Patients

Patients with either C3G (n=589) or aHUS (n=448) referred to the Molecular Otolaryngology and Renal Research Laboratories (MORL) from 2013-’19 for FHAA testing were included in this study. Serum and plasma samples were collected using our standard operating procedure (SOP), aliquoted, and stored at -80°C prior to testing (21). Control sera and plasma (n=300) were collected using the same SOP. The study was approved by the Institutional Review Board of Carver College of Medicine at the University of Iowa.

### Anti-Factor H Autoantibody Assay

FHAAs were detected as previously described (22, 23). Briefly, purified human FH (Complement Technology Inc, Tyler, TX) was coated in 1X PBS (pH=7.4) at a concentration of 10 μg/mL on a 96-well micro-titer plate, which was then kept overnight at 4°C. After washing three times with 1X PBST (1X PBS containing 0.1% Triton-X), free reactive sites were blocked with Ultrablock (AbD Serotec, Raleigh, NC) for 30 min at room temperature. Patient serum (1:50 dilution) was added for a 1-hour incubation at room temperature, after which plates were washed and incubated for another hour at room temperature with a horseradish peroxidase-labeled goat anti-human IgG antibody specific for the γ chain. After final washings, enzymatic activity was measured using OPD (o-phenylenediamine dihydrochloride) and absorbance was read at λ490.

A standard curve (4-parameter logistic regression) was generated for each run by serial dilutions of a positive sample (aHUS49, 3,000 arbitrary units (AU) at 1:50). The value was calibrated to a positive sample kindly provided by Dr. Marie Agnès Dragon-Durey (Georges Pompidou hospital, Paris, France).

### Epitope Mapping and Isotyping

To map binding epitopes, recombinant FH fragments of SCRs1-6, 6-8, 8-15, 15-18, 18-20 and mini-FH (1-4 and 19-20) were produced as previously described (3, 5, 24), and used as capturing/coating proteins in the aforementioned protocol. Similarly, recombinant FHR1, 2, and 5 with 6X HIS tag were used as capturing/coating proteins for testing FHAA cross-reactivity to FHR1, 2 or 5.

To determine IgG subclass and light chains, the protocol was repeated with mouse anti-human IgG1, IgG2, IgG3, IgG4, kappa and lambda antibodies (all from Millipore Sigma) used at a dilution of 1:1,000–1:2,000 as detecting antibodies.

### Other Autoantibody Detection

FB autoantibodies (FBAAs) were measured by ELISA and C3/C4/C5 nephritic factors were measured by cell-based hemolytic methods as previously described (25, 26).

### M-Protein Detection

All FHAA-positive patients were screened for M-proteins using serum protein electrophoresis and immunofixation electrophoresis (IFE) on a SPIFE Touch System (Helena Laboratories, Beaumont, TX).

### Complement Assays

Serum levels of C3 were measured by ELISA (Hycult Biotech Inc., Uden, Netherlands). C4 was measured using radial immunodiffusion (The Binding Site Inc., Birmingham, UK). Soluble C5b-9 and FH levels were measured using ELISA kits (Quidel Corporation, San Diego, CA).

### Genetic Analysis

Genomic DNA was extracted from peripheral blood using the Gentra Puregene Kit (Qiagen Inc., Valencia, CA) and integrity was evaluated by 1% agarose gel electrophoresis. Absorbance at 230:260:280 was measured using a NanoDrop 1000 spectrophotometer (Thermo Fisher Scientific, Wilmington, DE) to ensure DNA samples met quality metrics of 1.8 for 260/280 and 260/230 ratios. DNA concentration was determined using the Qubit dsDNA HS Assay Kit (Life Technologies, Carlsbad, CA). Samples were then screened using the Genetic Renal Panel focused on complement gene abnormalities, as previously described (27). To interrogate the *CFH-CFHR* region, multiplex ligation-dependent probe amplification (MLPA) was performed using MRC Holland SALSA kit (Amsterdam, Netherlands) and in-house designed probes (28).

### Western Blotting for FHR1

Serum or plasma (1:40 diluted) in Laemmli buffer was separated on 4–15% gel followed by in-house produced polyclonal rabbit antibodies to the first SCR of FHR1 and FHR2.

### Statistical Analyses

Statistical analysis was performed using GraphPad (version 8.2). The Student *t*-test or Mann-Whitney *U* Test was used to compare groups. *P* < 0.05 was considered statistically significant.

## Results

### FHAAs in C3G and aHUS

**Figure 1:**
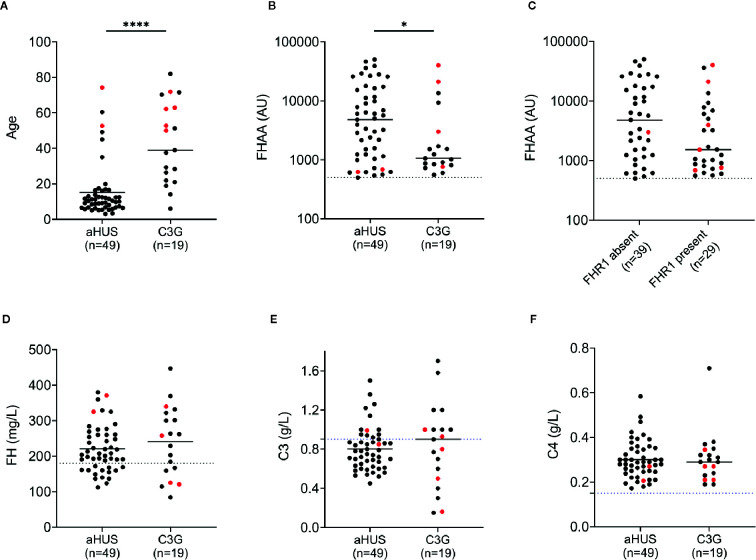
FH autoantibodies (FHAAs) and key biomarkers at the age-of-onset of disease in patients with aHUS and C3G. Patient with M- proteins are in red. **(A)** Age; **(B)** FHAA titers in aHUS and C3G; **(C)** FHAAs (C3G and aHUS) with and without FHR1; **(D)** FH levels; **(E)** C3 levels; **(F)** C4 levels. Solid lines: medians. Dashed lines: normal cutoff based on results from 300 healthy individuals. *P < 0.05 and ****P < 0.0001 by Mann-Whitney *U* test.

Nineteen patients (3.2%) in the C3G cohort and 49 patients (10.9%) in the aHUS cohort were positive for FHAAs. In both cohorts, both genders were equally affected (**Tables 1** and **2**), however FHAA-positive aHUS patients were significantly younger than FHAA-positive C3G patients (median age, 10.2 vs 38.3, respectively; *P* < 0.001, **Figure 1A**). FHAA titers were also significantly higher in patients with aHUS as compared to patients with C3G (median, 4787 AU *vs* 1149 AU, respectively; *P* < 0.05, **Figure 1B**).

**Table 1:** Demographic and genetic data for aHUS patients.

Patient	Sex	Age range	Ethnicity	Genetic findings	Copies		
aHUS				(rare variant MAF<0.01%)	*CFHR3*	*CFHR1*	*CFHR4*
aHUS1	M	6–10	Caucasian	N/A	N/A	N/A	N/A
aHUS2	M	6–10	Caucasian	no variants	0	0	2
aHUS3	M	11–15	Caucasian	no variants	0	0	2
aHUS4	M	6–10	Caucasian	no variants	1	0	1
aHUS5	F	6–10	Caucasian	N/A	0	0	2
aHUS6	M	6–10	Caucasian	no variants	0	0	2
aHUS7	F	16–20	Caucasian	no variants	0	0	2
aHUS8	F	1–5	Caucasian	no variants	0	0	2
aHUS9	F	6–10	Caucasian	N/A	N/A	N/A	N/A
aHUS10	M	16–20	African American	no variants	0	0	2
aHUS11	M	31–35	Caucasian	no variants	0	0	2
aHUS12	F	6–10	Caucasian	no variants	0	0	2
aHUS13	M	1–5	African American	*CFHR5* c.427A>C, p.Thr143Pro	0	0	2
aHUS14	F	6–10	Hispanic	no variants	1	0	1
aHUS15	M	11–15	Caucasian	no variants	0	0	2
aHUS16	M	11–15	Asian	no variants	0	0	2
aHUS17	F	11–15	Hispanic	no variants	0	0	2
aHUS18	M	6–10	Caucasian/African American	no variants	0	0	2
aHUS19	F	6–10	African American	no variants	0	0	2
aHUS20	M	6–10	Hispanic	N/A	N/A	N/A	N/A
aHUS21	F	6–10	Caucasian	*CFH* c.3644G>A, p.Arg1215Gln	0	0	2
aHUS22	M	11–15	Caucasian	no variants	0	0	2
aHUS23	M	16–20	Caucasian	no variants	0	0	2
aHUS24	M	61–65	Caucasian	no variants	1	0	1
aHUS25	F	11–15	Caucasian	no variants	0	0	2
aHUS26	M	11–15	Arabic	no variants	0	0	2
aHUS27	M	11–15	Caucasian	no variants	1	0	1
aHUS28	M	1–5	Caucasian	no variants	0	0	2
aHUS29	F	11–15	Caucasian	no variants	1	0	1
aHUS30	M	6–10	Hispanic	no variants	0	0	2
aHUS31	F	6–10	Caucasian	no variants	0	0	2
aHUS32	F	41–45	Caucasian	no variants	1	0	1
aHUS33	F	16–20	African American	no variants	0	0	2
aHUS34	F	6–10	Asian	no variants	0	0	2
aHUS35	F	11–15	Asian	N/A	0	0	2
aHUS36	M	6–10	Hispanic	no variants	0	0	2
aHUS37	M	6–10	Caucasian	no variants	0	0	2
aHUS38	M	1–5	African American	no variants	1	1	2
aHUS39	M	46–50	Caucasian	*CFH* c.3536T>C, p.Ile1179Thr	2	2	2
aHUS40	M	51–55	Caucasian	no variants	2	2	2
aHUS41	M	16–20	Hispanic	no variants	2	2	2
aHUS42	F	6–10	Hispanic	no variants	2	2	2
aHUS43	F	6–10	Caucasian	no variants	2	2	2
aHUS44	F	11–15	Asian	no variants	2	2	2
aHUS45	F	6–10	Hispanic	no variants	2	1	1
aHUS46	M	71–75	Caucasian	no variants	1	1	2
aHUS47	M	16–20	Caucasian	no variants	2	2	2
aHUS48	M	1–5	Arabic	no variants	2	2	2
aHUS49	F	1–5	Caucasian	no variants	N/A	N/A	N/A

N/A, not available.

**Table 2:** Demographic and genetic data for C3G patients.

Patient	Sex	Age range	Ethnicity	Genetic findings	Copies		
C3G				(rare variant MAF<0.01%)	*CFHR3*	*CFHR1*	*CFHR4*
C3G1	M	61–65	Caucasian	no variants	1	0	1
C3G2	M	26–30	Caucasian	no variants	1	1	2
C3G3	F	71–75	Caucasian	no variants	2	2	2
C3G4	M	26–30	Caucasian	N/A	N/A	N/A	N/A
C3G5	F	71–75	Asian	*C3* c.3214C>T, p.Arg1072Trp	2	2	2
C3G6	F	46–50	Caucasian	no variants	2	2	2
C3G7	F	51–55	Caucasian	no variants	3	3	2
C3G8	F	21–25	Caucasian	no variants	2	2	2
C3G9	M	36–40	Caucasian	no variants	2	2	2
C3G10	M	81–85	Caucasian	no variants	2	2	2
C3G11	M	66–70	Caucasian	no variants	2	2	2
C3G12	F	61–65	Caucasian	no variants	2	2	2
C3G13	F	6–10	Caucasian	*CFH* c.1854A>G, p.Asp619Gly	2	2	2
C3G14	M	21–25	Hispanic	no variants	2	2	2
C3G15	F	26–30	Caucasian	N/A	N/A	N/A	N/A
C3G16	M	11–15	Hispanic	N/A	N/A	N/A	N/A
C3G17	M	16–20	Hispanic	no variants	2	2	2
C3G18	F	36–40	Caucasian	no variants	0	0	2
C3G19	F	51–55	Caucasian	N/A	N/A	N/A	N/A

N/A, not available.

### FHAA Targeting Epitopes and FHR1 Deficiency

FHR1 deficiency as determined by either MLPA to detect homozygous deletion of the *CFHR1* gene or Western blotting to detect absence of the FHR1 protein was observed in 37 of 49 (76%) aHUS patients and 2 of 19 (11%) C3G patients (**Tables 3** and **4**).

**Table 3:** FHAAs in patients with atypical hemolytic uremic syndrome.

Patient	FHR1^^^	FHAA (AU)	FH Epitope					Mini-FH	FHR1	FHR2	M-Spike	FHAA	
			1–6	6-8	8–15	15–18	18–20					IgG subclasses	Light chains
aHUS1	absent	2875					+	+				IgG3	λ
aHUS2	absent	2140					+	+				IgG3	λ
aHUS3	absent	540					+	+				IgG3	k
aHUS4	absent	9828					+	+				IgG3	λ
aHUS5	absent	500					+	+				IgG3	λ
aHUS6	absent	724					+	+				IgG3	λ
aHUS7	absent	618					+	+				IgG3	λ
aHUS8	absent	3362					+	+	+			IgG1	λ
aHUS9	absent	25690					+	+	+			IgG3	λ
aHUS10	absent	4787					+	+	+			IgG1+IgG3	k
aHUS11	absent	2180					+	N/D	+			IgG3	λ
aHUS12	absent	17290					+	+	+			IgG3	k
aHUS13	absent	11220					+	+	+			IgG1+IgG3	λ
aHUS14	absent	1230					+	N/D	+			IgG3	λ
aHUS15	absent	609					+	+	+			IgG3	k
aHUS16	absent	46180					+	+	+			IgG1+IgG3	λ+k
aHUS17	absent	27990					+	+	+			IgG3	λ+k
aHUS18	absent	29120					+	+	+			IgG3	λ
aHUS19	absent	39000					+	+	+			IgG3	k
aHUS20	absent	1041					+	N/D	+			IgG3	λ+k
aHUS21	absent	1148					+	+	+			IgG3	λ
aHUS22	absent	1230					+	+	+			IgG3	k
aHUS23	absent	6041					+	+	+			IgG3	λ
aHUS24	absent	5640					+	+	+			IgG3	λ
aHUS25	absent	2401					+	+	+			IgG1	λ
aHUS26	absent	25750					+	+	+			IgG3	λ+k
aHUS27	absent	6361					+	+	+			IgG3	λ
aHUS28	absent	26450					+	+	+			IgG3	λ
aHUS29	absent	15220					+	+	+			IgG3	λ
aHUS30	absent	9018					+	+	+			IgG3	k
aHUS31	absent	50070					+	+	+	+		IgG3	λ
aHUS32	absent	11680	+				+	+	+			IgG1+IgG3+IgG4	λ+k
aHUS33	absent	1505	+				+	N/D	+			IgG1+IgG3	λ+k
aHUS34	absent	1571	+	+			+	+	+			IgG1+IgG3	λ
aHUS35	absent	16360	+	+	+		+	+	+			IgG3	λ+k
aHUS36	absent	17830			+		+	+	+			IgG3	λ
aHUS37	absent	622			+		+	N/D	+			IgG1+IgG3	λ
aHUS38	present	3217	+					+				IgG3	λ+k
aHUS39	present	621	+					+				IgG3	λ
aHUS40	present	3948			+						IgG λ	IgG1	λ
aHUS41	present	5019					+	+				IgG3	k
aHUS42	present	35990					+	+				IgG3	k
aHUS43	present	562					+	N/D				IgG3	k
aHUS44	present	6895					+	+				IgG1	k
aHUS45	present	7823					+	+				IgG3	k
aHUS46	present	683					+	+			IgG k	IgG1	k
aHUS47	present	986	+	+			+	+				IgG1	k
aHUS48	present	6053	+				+	+	+			IgG1+IgG3	λ+k
aHUS49	present	3000	+	+			+	+	+			IgG3	λ+k

^^^Determined by MLPA or the Western if no DNAs.

Blank, Negative; N/D, not done.

**Table 4:** FHAAs in patients with C3 glomerulopathy.

Patient	FHR1^^^	FHAA (AU)	FH Epitope					Mini-FH	FHR1	FHR2	M-Spike	FHAA	
			1-6	6-8	8-15	15-18	18-20					IgG subclasses	Light chains
C3G1	absent	2990	+					+			IgG k	IgG1	k
C3G2	present	9315	+					+				IgG1+IgG4	λ+k
C3G3	present	1059	+					+				IgG4	λ
C3G4	present	13550	+					+				IgG1+IgG3	λ
C3G5	present	21010	+					+			IgG k	IgG1	k
C3G6	present	1514		+							IgG λ	IgG1	λ
C3G7	present	39980		+							IgG λ	IgG3	λ
C3G8^#^	present	555	+	+				+				IgG3	k
C3G9	present	809	+	+				+				IgG3	k
C3G10	present	1017			+							IgG1	λ
C3G11	present	869	+	+	+		+	+				IgG1	λ+k
C3G12	present	759	+	+			+	+			IgG k	IgG3	k
C3G13*	present	1525					+	+				IgG3	k
C3G14	present	601					+	+				IgG3	k
C3G15	present	725					+	+				IgG3	λ
C3G16	present	1697		+			+	+	+	+		IgG3	λ
C3G17	present	1238					+	+	+	+		IgG3	k
C3G18*	absent	843					+	N/D	+			IgG3	k
C3G19	present	908	+				+	+	+			IgG3	k

^^^Determined by MLPA or the Western if no DNAs.

*C3G13 and C3G18 are positive for C3Nefs.

^#^C3G8 is also positive for FBAAs.

Blank, negative; N/D, not done.

In aHUS, FHAA titers were higher in patients deficient as compared to patients replete in FHR1 (median 5,841 vs 3,217, respectively) although the difference was not statistically significant (*P*=0.131, Mann-Whitney *U* Test, **Figure 1C**). 37/37 (100%) of aHUS patients deficient in FHR1 carried FHAAs that primarily targeted the C-terminus of FH. In four-fifths of these patients (30/37, 81%), the FHAAs cross-reacted with FHR1, while in one patient (aHUS31), cross-reactivity to FHR2 was also seen. In these patients, the addition of recombinant FHR1 blocked binding of FHAAs to FH. Multiple epitopes of FH were recognized in six of the 37 FHR1-deficient aHUS patients, including four patients who were co-positive for FHAAs against N-terminus SCRs and two patients who were co-positive for FHAAs against mid-SCRs 8-15.

Of the 12 aHUS patients who express FHR1, six (aHUS41-46) had FHAAs that bind to the C-terminus of FH only, but in none of these patients did the FHAAs show cross-reactivity with FHR1. Two patients (aHUS38, 39) had FHAAs that bind to the N-terminus of FH only, while three patients (aHUS47-49) were co-positive for FHAAs that recognized N- and C-terminal epitopes of FH. In two of these three patients, there was cross-reactivity with FHR1. In one patient (aHUS40), FHAAs recognized an epitope in SCRs 8-15 of FH.

Only two patients with C3G were FHR1 deficient. In one patient (C3G1), FHAAs targeted the N-terminus (SCRs1-6) alone while in the other patient (C3G18), FHAAs reacted with the C-terminus of FH and also cross-reacted with FHR1. Of the 17 other C3G patients, all of whom express FHR1, 16 had FHAAs that recognized specific FH epitopes: four patients (C3G2-5) had FHAAs that bind to FH SCRs 1-6; two patients (C3G6, 7) had FHAAs that bind to FH SCRs 6-8; two patients (C3G8, 9) had FHAAs to both fragments; one patient (C3G10) had FHAAs that bind to FH SCRs 8-15; and four patients (C3G13-15, 17) had FHAAs that bind to SCRs 19 and 20. There were three patients (C3G12, 16, 19) whose FHAAs reacted with both N- and C- terminal SCRs of FH.

No patients in this study carried FHAAs that recognized FH SCRs15-18 or had cross reactivity to FHR5.

### IgG Subclasses and M-Proteins

The distribution of IgG subclasses was similar in the two disease cohorts and is listed in **Tables 3** and **4**. In the aHUS cohort, the prominent subclass was IgG3 (35/49, 71%), with most patients (29/35, 83%) having a restriction of either lambda or kappa, although six patients (6/35, 17%) were co-positive for lambda and kappa. Another six patients (6/49, 12%) were positive for only IgG1 with either lambda or kappa restriction; two of these patients had MGRS (one each of IgG κ and λ). Seven patients (7/49, 14%) were co-positive for IgG1 and IgG3 and one patient (1/49, 2%) was co-positive for IgG1, IgG3 and IgG4. No patient was positive for IgG2. With respect to light chains, 25 patients (25/49, 51%) were positive for lambda only, 14 patients (14/49, 29%) for kappa only, and 10 patients (10/49, 20%) for both.

In the C3G cohort, the prominent subclass was also IgG3 with either lambda or kappa restriction (11/19, 58%). There were two patients with MGRS in this group (one each of IgG κ and λ). Five patients (5/19, 26%) were positive for IgG1, with four patients showing lambda or kappa restriction, consistent with subclasses of M-spikes found in three patients (two IgG κ and one IgG λ); one patient was positive for both light chains. One patient (1/19, 5%) showed co-positivity for IgG1 and IgG3 with lambda restriction and one patient (5%) showed co-positivity for IgG1 and IgG4 with reactivity to both light chains. One patient (1/19, 5%) was positive for IgG4 only with kappa restriction. No patient was positive for IgG2.

### Other Acquired Drivers of Disease

No other autoantibodies were detected in aHUS patients positive for FHAAs however one patient in the C3G cohort was co-positive for FBAA (C3G8) and two patients had C3 nephritic factors (C3G13, 18) (**Table 4**).

### Complement Dysregulation With FHAAs

Low plasma FH levels were detected in 14 of 48 (29%) and 6 of 19 (32%) patients with aHUS and C3G, respectively (**Figure 1D**). There was no correlation between FH levels and FHAA titers. C3 levels were low in 12 of 20 (60%) patients with aHUS and 7 of 16 (44%) patients with C3G (**Figure 1E**). C4 levels were normal in all patients (**Figure 1F**). Soluble C5b-9 was elevated in 15 of 19 patients (79%) with aHUS not on Eculizumab and in 11 of 16 C3G patients (also not on Eculizumab; 69%), consistent with uncontrolled activity of the terminal complement pathway.

### Genetic Findings

Genetic testing was completed in 45 aHUS and 15 C3G patients. Ultra-rare genetic variants (defined as a minor allele frequency (MAF) <0.01% and resulting in a nonsynonymous amino acid change) were identified in five patients (**Tables 1** and **2**). Three patients carry rare variants in the *CFH* gene (patients aHUS21, 39 and C3G13). The variants found in the two aHUS patients were in SCR20 of FH. One, FH p.R1215Q (in aHUS21), affects surface regulation by impairing surface heparin binding (3, 29); for the second variant, FH p.Ile1179Thr (in aHUS39), functional data are unavailable. The C3G patient (C3G13) carries FH p.Asp619Gly in SCR10, again a variant for which functional data are lacking. Another C3G patient (C3G5) carries C3 p.Arg1072Trp. In addition, one aHUS patient (aHUS13) carries a rare variant of unknown significance in the *CFHR5* gene.

## Discussion

Herein, we report a retrospective study of FHAAs in aHUS and C3G patient cohorts from North America. Overall, FHAAs were found in 10.9% of aHUS and 3.2% of C3G patients, respectively, consistent with prior reports in populations of European decent (13, 30). The high prevalence of FHR1 deficiency in association with FHAAs is seen only in the aHUS cohort and not in the C3G cohort (**Tables 3** and **4**).

In more than 80% of patients with FHR1 deficiency, FHAAs bind to both the carboxy-terminus of FH and FHR1. In these patients, FHAA titers tend to be extremely high in the acute phase of disease (**Figure 1B**, data collected in acute phase) but drop during remission, suggesting that the presence of FHR1 plays an important role in suppressing auto-immunogenicity of FH when a trigger is present. Consistent with this hypothesis, in these patients recombinant FHR1 can compete off FH for FHAA binding.

The last two SCRs of FH are essential for self-surface recognition and have ligand-binding sites for heparan sulfate, sialic acid and the complement cleavage product, C3d. Importantly, sequence homology between SCRs 19 and 20 of FH and SCRs 4 and 5 of FHR1 is very high, with amino acid differences only at two positions (S1191 and V1197 on FH *vs* L290 and A296 on FHR1) (31). It has been postulated that subtle conformational changes at residues 1,182–1,189 (due to S1191) in FH SCR20 occur during infections and may be auto antigenic in the absence of FHR1 (18). Our data appear to support this hypothesis since >92% of FHAA patients with FHR1 deficiency were pediatric cases with a median age of 10 (IQR 6.9–12.7), suggesting that the development of FHAAs is associated with common school-related infections.

The consequence of FHAAs that impair C-terminal function of FH is dysregulation of complement control on host cell surfaces. FHR1 deficiency alone, however, is not sufficient to trigger the generation of FHAAs. The deletion of 79.4 kb on chromosome 1 (gnomAD ID: MCNV_1_81) that includes two *CFH*-related genes, *CFHR3* and *CFHR1*, is a common CNV in the human genome with homozygous deletion of both copies of the *CFHR3-CFHR1* genes present in 4.1% of Europeans, 16.2% of Africans, 1.9% of Latinos and 0.3% of East Asians (data based on the reported non-diploid CN frequency in the gnomAD). In fact, FHR1 deficiency has been reported in FHAA-*negative* aHUS patients at a frequency that is higher than that in an ethnically matched control population (32).

In our aHUS cohort, in contrast, after excluding patients with FHAAs, the frequency of FHR1 deficiency was comparable to that found in a control population of European decent (nine of 255 patients, 3.5%). In addition, we commonly detected FHAAs in patients who express *CFHR1.* Of the 29 patients with FHR1 who were positive for FHAAs, there were 12 cases of aHUS (24% of all FHAA-positive cases of aHUS) and 17 cases of C3G (89% of all FHAA-positive cases of C3G). In about half of these cases, the antibody recognized carboxy-terminal SCRs of FH; however, in no case was there cross-reactivity to FHR1 and the addition of recombinant FHR1 *ex vivo* has no influence on FHAA binding results.

In seven of 19 (37%) C3G patients (C3G1-5, 8, 9) and two of 49 (4%) aHUS patients (aHUS38, 39) with FHR1, the FHAAs reacted only with SCRs at the N-terminus (SCRs1-6). Epitope reactivity was confirmed using mini-FH (SCRs1-4+19-20). The N-terminal SCRs1-4 is the site of DAA and CA. Blocking these two major regulatory functions of FH would be predicted to lead to complement dysregulation in the fluid phase as well as on cell surfaces. Why some patients develop a C3G phenotype and others develop an aHUS phenotype is not clear but may reflect differences in the degree of residual DAA or CA, as well as factors that determine complement control in local microenvironments like the glomerulus. We speculate that in general C3G patients have better control of AP activity on cell surfaces as compared to aHUS patients due to the presence of complement regulators (*i.e.* CD46, CD55) or due to FH itself. In this study, for example, we identified two aHUS patients (aHUS21, 39) carrying pathogenic or likely pathogenic variants in *CFH* as genetic drivers of disease. With ongoing dysregulation primarily in the fluid phase, C3G patients present with chronic phenotypes such as proteinuria and hematuria, while aHUS patients present with acute phenotypes like endothelial cell damage and complement-mediated coagulopathy.

None of the aHUS patients had other acquired drivers of disease while in C3G, two patients had C3Nefs and one patient had FBAAs (*P* < 0.05 by Fisher exact test). This finding suggests that while aHUS patients are more likely to have FHAAs as a sole acquired driver, C3G patients may be co-positive for other acquired drivers of disease such as nephritic factors, perhaps implying that FHAAs play a secondary role in C3G.

Interestingly, in 37 of 49 (76%) aHUS patients and 16 of 19 (84%) C3G patients, the circulating FHAAs demonstrated monoclonal characteristics (one subclass of heavy chains + one type of light chains) with IgG3 followed by IgG1 being the most common heavy chain isotypes. These data are consistent with most previous reports (9, 33–35), but are at odds with the findings described by Guo *et al.*, who showed that FHAAs in the Chinese population are typically polyclonal (36). The presence of the lambda light chain was more dominant in the aHUS cohort while both light chains had equal presence in the C3G cohort.

Albeit many FHAAs appeared monoclonal, we only observed seven M-proteins by IFE (the most sensitive method for detecting circulating M-proteins) in both cohorts, two aHUS patients (aHUS40, 46) and five C3G patients (C3G1, 5–7, 12). These patients were all over 50 years of age and while monoclonal gammopathy has emerged as an underlying cause of C3G in the elderly (13, 14, 37), its association with aHUS has not been previously reported.

MGRS is a recently defined disease entity, in which the underlying pathogenesis for the renal disease is associated with circulating monoclonal immunoglobulins or M-proteins that drive renal injury. The malignant clonal B cell clones do not meet criteria for overt multiple myeloma/B-cell proliferation. M-proteins can directly deposit in the glomeruli and activate complement through the classical pathway resulting in immune complex glomerulonephritis, or alternatively directly activate the AP in the fluid phase or block complement regulators resulting in M-protein induced C3G or aHUS (13, 14, 19, 20).

As compared to C3G (5/19, 26%), the prevalence of M-proteins in aHUS (2/49, 4%) is rare. Patient aHUS46 has a monoclonal IgG1 kappa directed against the C-terminus of FH (without FHR1 reactivity) that impairs the surface regulation of the AP, consistent with aHUS phenotype. However, in patient aHUS40, FHAAs (also IgG1 but lambda light chain) only bind to the mid portion of FH (SCRs 8–15). In this patient, plasma FH is normal, C3 is borderline low, and sC5b-9 is slightly elevated, findings consistent with ongoing complement dysregulation. Interestingly, C3G patient C3G10 is also positive for FHAAs that target only SCRs 8-15 of FH with no apparent M-proteins and has a similar biomarker profile (normal FH, borderline low C3, slightly elevated sC5b-9). Additional research is warranted in these two cases, as it would be of great interest to clarify the underlying mechanisms by which FHAAs that target the mid-portion of FH impact complement control.

Finally, two C3G patients (C3G6, 7) circulate monoclonal FHAAs (IgG1, kappa and lambda, respectively) that target only SCR7 of FH. Recently, Li, et al. described a Chinese C3G patient with FHAAs that bind to SCR7. Functional studies showed that in this patient, the SCR7-recognizing FHAAs inhibited FH binding to C3b and accelerated formation of C3 convertase (37). In addition, FHL-1, a truncated version of FH containing the first 7 SCRs of FH, has regulatory activity that involves domains SCRs5-7 (38). Thus, compromising SCR7 function may play a role in the pathogenesis of MGRS-C3G.

**Figure 2:**
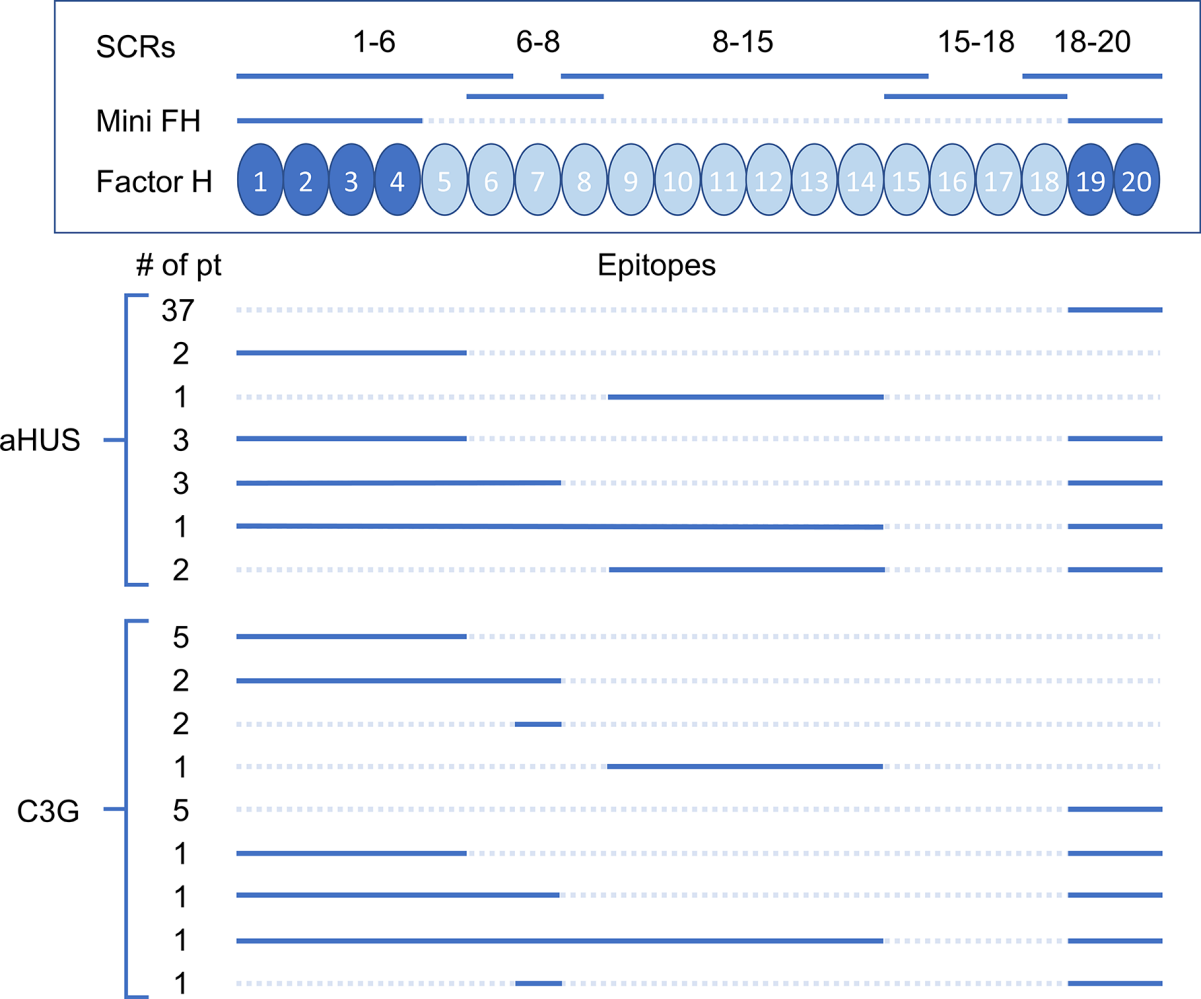
Epitopes of factor H autoantibodies (FHAAs) targeting domains on factor H (FH). Recombinant FH fragments (SCRs) and the mini FH construct are shown above a schematic of FH. Below are shown the epitope mapping results for aHUS (n=49) and C3G (n=19) patients (solid lines = positive FHAA results, dash lines = negative FHAA results).

In conclusion, we provide a comprehensive analysis of FHAAs in patients with aHUS and C3G (**Figure 2**). In aHUS, the absence of FHR1 is associated with a high incidence of FHAAs in patients age under 20 years of age; in patients over 50 years of age, FHAAs may be associated with MGRS. In C3G, FHAAs are more likely to be co-positive with other autoantibodies and the likelihood of MGRS in older patients is higher. Our data highlight the value of epitope mapping and isotyping in patients who are positive for FHAAs as a method of refining the underlying pathophysiology of complement dysregulation in the fluid phase and/or on cell surfaces.

## Data Availability Statement

The datasets generated for this study are available on request to the corresponding author.

## Ethics Statement

The studies involving human participants were reviewed and approved by Institutional Review Board of Carver College of Medicine at the University of Iowa. Written informed consent to participate in this study was provided by the participants’ legal guardian/next of kin.

## Author Contributions

YZ and RS designed the research, analyzed and interpreted data, and wrote the manuscript. NG, DS, AD, MJ, NM, GP, and AT performed experiments and participated in data analysis. CN and CS provided crucial conceptual input. CS contributed essential reagents. All authors contributed to the article and approved the submitted version.

## Funding

Supported in part by National Institutes of Health R01 DK110023.

## Conflict of Interest

The authors declare that the research was conducted in the absence of any commercial or financial relationships that could be construed as a potential conflict of interest.
